# P04.06. Complementary and alternative medicine in the treatment of pain in fibromyalgia: a systematic review of randomized controlled trials

**DOI:** 10.1186/1472-6882-12-S1-P276

**Published:** 2012-06-12

**Authors:** L Terhorst, M Schneider

**Affiliations:** 1 University of Pittsburgh, Pittsburgh, USA

## Purpose

The purpose of this study was to systematically review the literature for randomized trials of complementary and alternative medicine (CAM) interventions for fibromyalgia (FM).

## Methods

A comprehensive literature search was conducted. Databases included the Cochrane library, PubMed, PsycINFO, Cumulative Index to Nursing and Allied Health, Natural Medicines Comprehensive Database, Manuals of Alternative and Natural Therapy Index System (MANTIS), Index for Chiropractic Literature, and Allied and Complementary Medicine (AMED). Inclusion criteria were: (1) subjects were diagnosed with fibromyalgia and (2) the study design was a randomized controlled trial that compared a CAM therapy vs a control group. Studies were subgrouped by CAM treatment into 11 categories. Evidence tables and forest plots were organized to display quality ratings and effect sizes of each study.

## Results

The literature search yielded 1722 results; 102 abstracts were selected as potential articles for inclusion. Sixty studies met inclusion criteria and were rated by two reviewers; 18 were rated as good quality; 20 moderate quality; 18 low quality; and 4 very low quality. Synthesis of information for CAM categories represented by more than five studies revealed that balneotherapy and mind-body therapies were effective in treating FM pain. This study analyzed recent studies and focused exclusively on randomized controlled trials. Despite common use of manual therapies such as massage and manipulation to treat patients with FM, there is a paucity of quality clinical trials investigating these particular CAM categories.

## Conclusion

Most of these studies identified were preliminary or pilot studies, thus had small sample sizes and were likely underpowered. Two CAM categories showed the most promising findings, balneotherapy and mind-body therapies. Most of the other CAM categories showed a trend favoring the treatment group. It appears that several CAM therapies show some preliminary treatment effect for FM pain, but larger trials that are more adequately powered are needed.

